# Incidence, Risk Factors, and Outcomes of Arterial Thromboembolism in Patients with Pancreatic Cancer Following Palliative Chemotherapy

**DOI:** 10.3390/cancers10110432

**Published:** 2018-11-12

**Authors:** Yu-Shin Hung, Jen-Shi Chen, Yen-Yang Chen, Chang-Hsien Lu, Pei-Hung Chang, Wen-Chi Chou

**Affiliations:** 1 Division of Hematology-Oncology, Department of Internal Medicine, Chang Gung Memorial Hospital at Linkou and Chang Gung University College of Medicine, Taoyuan 333, Taiwan; f22338@cgmh.org.tw (Y.-S.H.); js1101@cgmh.org.tw (J.-S.C.); 2 Division of Hematology-Oncology, Department of Internal Medicine, Chang Gung Memorial Hospital at Kaohsiung, Kaohsiung 833, Taiwan; chen.y9964@gmail.com; 3 Division of Hematology-Oncology, Department of Internal Medicine, Chang Gung Memorial Hospital at Chiayi, Chiayi 612, Taiwan; luchanghsien@gmail.com; 4 Division of Hematology-Oncology, Department of Internal Medicine, Chang Gung Memorial Hospital at Keelung, Keelung 204, Taiwan; ph555chang@cgmh.org.tw

**Keywords:** pancreatic cancer, arterial thromboembolism, myocardial infarction, ischemic stroke, predictor

## Abstract

Background: Few studies have explored the association between pancreatic cancer and arterial thromboembolism (aTE). Methods: A total of 838 consecutive patients receiving palliative chemotherapy for pancreatic cancer between 2010 and 2016 were retrospectively enrolled. The clinical characteristics of patients were analyzed to determine the incidence, risk factors, and survival outcome of aTE in patients with pancreatic cancer. Results: aTE occurred in 42 (5.0%) of 838 patients. Patients with aTE had a worse survival outcome than those without (5.1 months versus 7.8 months, hazard ratio 1.53, 95% confidence interval [CI]: 1.12–2.09). Stage IV disease, high aspartate transaminase level, and comorbidity with hypertension or atrial fibrillation were four independent predictors of aTE. A concise predictive model stratified patients into low (0–1 predictor), intermediate (2 predictors), and high (3–4 predictors) risk groups. The hazard ratios for the comparison of patients in intermediate and high risk groups with those in low risk group were 4.55 (95% CI: 2.31–8.98), and 13.3 (95% CI: 5.63–31.6), respectively. Conclusion: Patients with pancreatic cancer undergoing palliative chemotherapy have an increased risk of aTE. A predictive model showed that patients presented with 3 or 4 predictors had the highest risk for developing aTE.

## 1. Introduction

Cancer is associated with hyper-coagulation status, which increases the risk of both venous and arterial thromboembolism (TE) [1,2]. The association between cancer and venous thromboembolism (vTE) has been widely emphasized and well-studied [3,4,5,6,7,8,9]; however, only a few studies have explored the relation of cancer and arterial thromboembolism (aTE) [10,11,12,13,14,15,16,17,18,19,20,21]. Because of the catastrophic consequences, occurrence of aTE in patients with cancer eventually results in the decline of physical function and quality of life, aggravating the burden of antitumor treatment, and increasing the risk of mortality [20,21,22].

An increasing number of studies are beginning to explore the association between pancreatic cancer and aTE [10,23,24]. However, due to the rarity of aTE and the inherent limitations imposed by the national registry data [24], data regarding the risk factors and the impact of aTE on survival outcome in patients with pancreatic cancer were limited. Palliative chemotherapy is the standard treatment for patients with unresectable or metastatic pancreatic cancer and unfortunately, the impact of chemotherapy on the overall incidence of aTE has not been thoroughly investigated. This study aimed to determine the incidence, risk factors, and survival outcome of aTE in patients with pancreatic cancer. Finally, we aimed to develop a concise predictive model for clinical utility to stratify the risk of aTE in patients with pancreatic cancer.

## 2. Results

### 2.1. Patient Demographics and Clinical Characteristics

The demographic characteristics of 838 patients are shown in Table 1. The median age was 62 years (range, 23–89 years) and 59.3% of patients were men. Majority of the patients (71.2%) had good ECOG PS 0–1, and 27.1% of the patients had no comorbidity. The most common comorbidities were hypertension (39.6%), diabetes (37.4%), and coronary artery disease (6.2%). In total, 78.2% of the patients had stage IV disease, and the three most common metastatic sites were liver (52.3%), peritoneum (28.5%), and distant lymph nodes (17.9%). The most common antitumor agent received by our patient cohort was gemcitabine (94.5%), followed by platins (51.7%), S-1 (40.0%), and 5-fluorouracil (30.7%). Less than 2% of our patients had been treated with erlotinib, irinotecan or nab-paclitaxel due to the lack of reimbursement by the national health insurance in Taiwan.

### 2.2. Patient Outcomes and the Impact of aTE on Overall Survival

**Figure 1:**
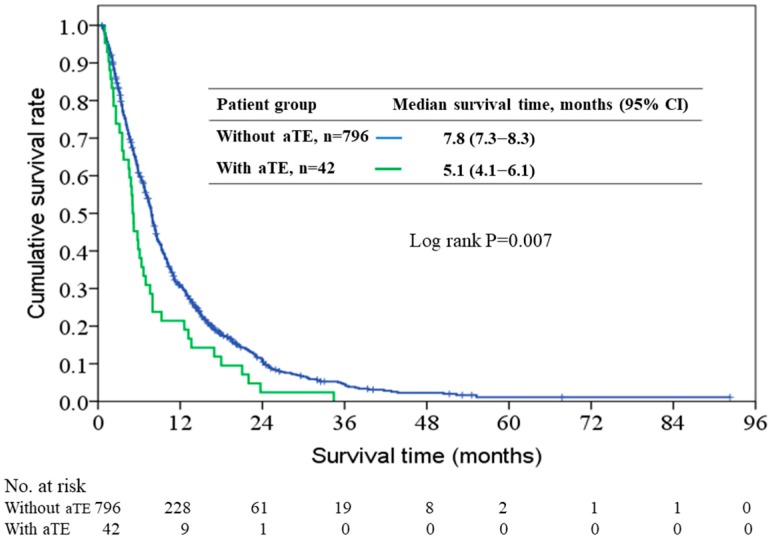
Kaplan–Meier overall survival curves for patients with and without arterial thromboembolism (aTE).

**Figure 2:**
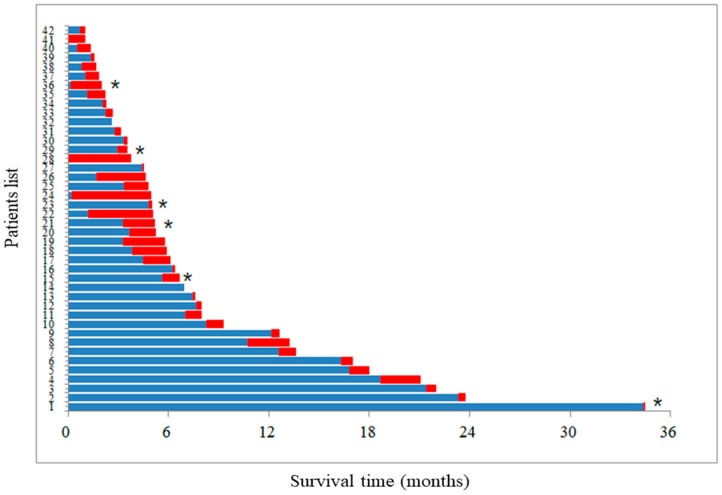
The time interval between the initiation of chemotherapy to aTE occurrence (blue color) and from aTE occurrence to patient’s death (red color). * indicates patients who received anticoagulants before aTE occurrence.

With a median overall survival time of 7.7 months (range, 0.6–55.6), 754 patients (90.0%) had died by the end of study period. aTE occurred in 42 (5.0%) of the 838 patients, including 36 patients (4.3%) with ischemic stroke and six patients (0.7%) with myocardial infarction. The median overall survival time was 5.1 months (95% CI, 4.1–6.1) and 7.8 months (95% CI, 7.3–8.3) for patients with and without aTE, respectively (Figure 1). Figure 2 shows the time interval between the events of initiation of chemotherapy, development of aTE, and patient’s death. All 42 patients that developed aTE had died by the end of study. The median time interval from initiation of chemotherapy to onset of aTE and the median time interval from aTE onset to death was 3.5 months (range, 0.1–34.4) and 0.8 months (range, 0–4.7), respectively. In total, six of the 42 patients received anticoagulation (either aspirin or clopidogrel) treatment. All six patients received antiplatelet as primary or secondary prevention for cardiac or stroke events based on underlying comorbidities. There were no significant differences of the median time interval from initiation of chemotherapy to aTE onset (3.32 vs. 3.39 months, *p* = 0.53) and from aTE onset to death (0.53 vs. 0.79 months, *p* = 0.62) between patients who received anticoagulation treatment and those who did not receive anticoagulation treatment.

In a univariate model for survival analysis, patients with aTE had a worse prognosis than those without aTE (hazard ratio [HR] 1.53, 95% CI: 1.12–2.09, *p* = 0.007). After being adjusted for gender, age, ECOG PS and CCI in multivariate analysis, aTE was still an independent poor prognosticator for overall survival (adjusted [HR] 1.41, 95% CI: 1.03–1.93, *p* = 0.031).

### 2.3. Risk Factors for aTE

**Figure 3:**
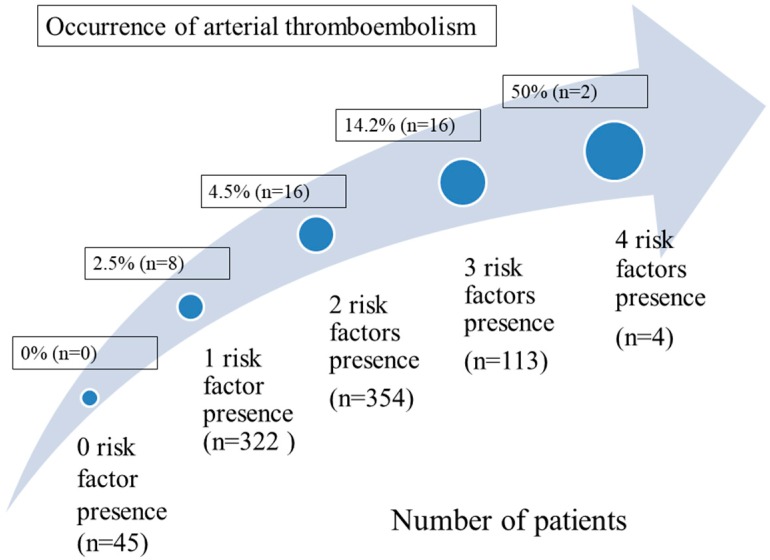
The distribution of aTE incidence according to numbers of risk factors present in patients.

Univariate and multivariate analyses of clinical variables to predict aTE occurrence are shown in Table 2. Univariate analysis identified BMI > 23 kg/m^2^, hypertension, cerebrovascular disease, atrial fibrillation (AF), stage IV disease, and aspartate transaminase (AST) level as significant predictors for the development of aTE. Only hypertension, AF, stage IV disease, and high AST level were independent risk factors in multivariate analysis. The distribution of aTE incidence according to numbers of risk factors present is showed in Figure 3. For patients who presented with 0, 1, 2, 3, and 4 risk factors, the aTE incidence rate was 0 % (0 of 45), 2.5% (8 of 322), 4.5% (16 of 354), 14.2% (16 of 113), and 50% (2 of 4), respectively.

### 2.4. Predictive Model of aTE Incidence Based on Presence of Numbers of Risk Factor

**Figure 4:**
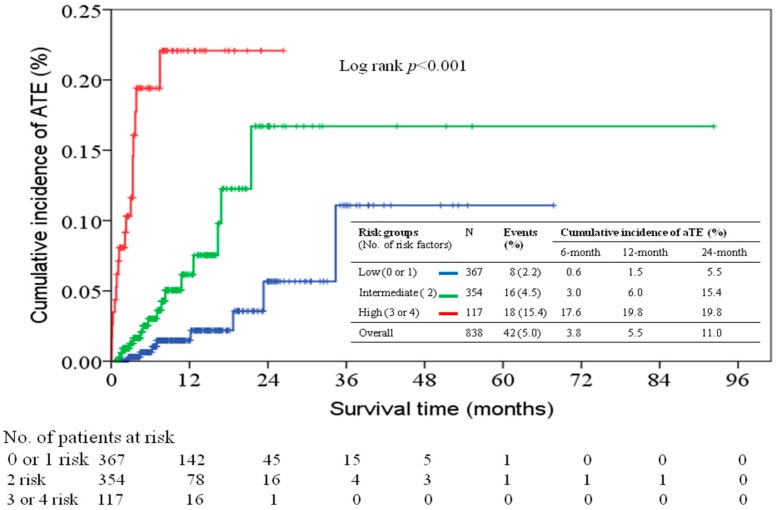
The cumulative incidences of development of aTE in patients in different risk groups.

All patients were categorized into low (0–1 risk factor), intermediate (2 risk factors), and high risk groups (3–4 risk factors) based on the number of risk factors presented by patients. The cumulative incidences of development of aTE in patients with different risk groups are shown in Figure 4. The 6-, 12-, and 24-months cumulative incidences were 0.6%, 1.5%, and 5.5% for the low risk group, respectively; 3.0%, 6.0%, and 15.4% for the intermediate risk group, respectively; and 17.6%, 19.8%, and 19.8% for the high risk group, respectively. The hazards ratios for the comparison of patients in the intermediate and high risk groups with those in the low risk group were 4.55 (95% CI: 2.31–8.98, *p* < 0.001), and 13.3 (95% CI: 5.63–31.6, *p* < 0.001), respectively.

## 3. Discussion

This is the first study to evaluate the impact of aTE in patients with pancreatic cancer who underwent palliative chemotherapy. In our patient cohort, aTE was detected in 5.0% of 838 patients. We identified that aTE was a poor prognostic factor for survival outcome, even after adjusting for other poor prognostic clinical variables. The median survival time was only 0.8 months between the aTE event and death in our cohort. Stage IV disease, high AST level, and comorbidity with hypertension or AF were the independent predictors for the development of aTE in patients with pancreatic cancer. Accordingly, we constructed a concise predictive model to predict the occurrence of aTE based on the number of the risk factor presented by the patients. The model could assist physicians in risk stratification of aTE in patients with pancreatic cancer and help to identify highest-risk patients who might benefit from prophylaxis with anticoagulation.

In contrast to the general concept of the association between vTE and pancreatic cancer [9,22,23], aTE is a less common but more devastating complication. The Memorial Sloan-Kettering Cancer Center (MSKCC) study revealed that 660 of the 1,915 patients (34.5%) experienced vTE whereas only 30 patients (1.6%) had aTE following being diagnosed with pancreatic cancer [23]. The MSKCC series incorporated aTE and vTE into TE for survival analysis based on 1915 patients. The author identified that the development of any TE significantly increased the risk of death, however, vTE accounted for 95.6% of all the TE, while only 4.5% were aTE. Therefore, the influence of TE on survival outcome was majorly contributed by vTE. Due to the disease rarity and heterogeneity of aTE from myocardial infarction to ischemic stroke, the incidence of aTE in patients with pancreatic cancer had not been thoroughly examined. Based on the present multicenter study with a relatively large number of events, we were able to analyze the impact of aTE on survival outcome among patients with pancreatic cancer who underwent palliative chemotherapy. In addition, we also analyzed the time sequences between the initiation of chemotherapy, development of aTE, and patient’s death. Several studies reported the peak incidence of ischemic stroke happened within first 6 months of cancer diagnosis [11,24] and the medial survival after ischemic stroke was around 1 to 4.5 months [15,16]. The peak incidence of aTE and survival outcome after the diagnosis of aTE was comparable with previous reports. Awareness of the survival impact of aTE and the time sequences for these major events might help physicians to counsel the appropriate timing for the prophylaxis of anticoagulation and provide appropriate end-of-life care after the occurrence of aTE.

Our results demonstrated a higher incidence of aTE (5.0%) in Taiwanese patients with pancreatic cancer than the Western population from the MSKCC series (1.6%) [23]. As advanced tumor stage significantly associated with aTE [23], the higher incidence of aTE in our series is possibly due to all our patient cohort having unresectable stage III or IV cancer whereas the MSKCC cohort enrolled patients a from stage I to stage IV. Furthermore, our study enrolled patients diagnosed with pancreatic cancer between 2010 and 2016 compared to the earlier patient cohort in the MSKCC series (2000 to 2009); there has been trend toward increased primary intervention therapy for cancer patients with myocardial infarction in modern years [25], which might allow more patients to confirm the diagnosis of coronary artery disease, therefore, partially contribute to the increase incidence of aTE in our patient cohort. Most of the studies [10,11] included myocardial infarction as one of the aTE events in cancer patients, though myocardial infarction were the result of atherosclerotic plaques. Excluding myocardial infarction as an aTE event, the incidence in our cohort is 4.3%, which is still higher than the MSKCC series. This further supported our theory that was because of the possible earlier intervention of the aTE events and later stages in our cohort.

Previous studies reported that various patient- [17,26,27], treatment- [11,12], and tumor-related factors [10,13,14] contribute to aTE in cancer patients. The current study identify that advanced tumor stage, high AST value, and comorbidity with hypertension and AF are the independent predictors of aTE in patients with pancreatic cancer who underwent palliative chemotherapy. AF is traditionally considered a risk factor for ischemic stroke in the general population [28] and it weighted the highest hazard ratio in multivariate analysis among the four independent predictors. Unfortunately, only 2 of 13 patients with AF received aspirin as stroke prophylaxis in our patient cohort. The current study highlighted the need for anticoagulation in pancreatic cancer patients with AF. Our study showed a predictive model in which combining AF and other risk factors could provide a risk stratification of cumulative incidence of aTE in patients with pancreatic cancer.

Our study included several laboratory parameters using the cutoff point of the median value in statistically analysis. Only AST was a significant predictor for aTE in both univariate and multivariate analysis. The cut-off value of AST in our report was 34 μ/L, which was the same to the upper limit of normal (ULN) in our institute. AST level was found to be associated with gender, alcohol consumption, serum lipid profiles and body mass index in normal population [29]. Therefore, AST approaching this level might be considered as clinically insignificant in medical care. The utility of using AST with more than 1-fold ULN in predicting aTE need further exploration and a prospective study is necessary to confirm the optimal cutoff value of AST in this model. Other parameters (hemoglobin, leukocyte count, and platelet count) present in the Khorana risk model [7], which were developed to predict vTE incidence in cancer patients who underwent palliative chemotherapy, were statistically insignificant. Although cancer is associated with a hypercoagulable state, with numerous reports of vTE and aTE as the initial manifestation or sequelae of cancer [10,11,12,13,14,15,16,17,18,19,20,21], our analysis raised the question of a possible distinct pathogenesis mechanism for aTE and vTE in cancer patients. For example, atherosclerosis might a possible pathogenic mechanism related to aTE in cancer patients [30]. These two devastating events should possibly be assessed separately to identify the highest-risk population among cancer patients.

Use of chemotherapy has been reported to be associated with a 6.5-fold greater risk of vTE in a population- based study [31]. One early study that included 10,963 Taiwanese cancer patients reported the incidence of ischemic stroke was 0.137% within one month after chemotherapy regardless cancer types [15]. Gemcitabine, 5FU, and platins are cytotoxic chemotherapeutic agents that are fundamental in treatment of pancreatic cancer [32]. Use of gemcitabine had been reported to increase the risk for vTE [33], whilst platins were the most commonly used chemotherapeutic agents in patients with cancer preceding ischemic stroke [15]. Our study did not identify the association of aTE and chemotherapeutic compounds in patients with pancreatic cancer. Because all patients had received chemotherapy treatment, we were unable to evaluate whether receiving chemotherapy increased the incidence of aTE.

The strength of our study was the large patient numbers from multiple centers in Taiwan. To the best of our knowledge, this is the first study to evaluate the incidence, risk factors, and survival outcome of aTE in patients with pancreatic cancer who underwent palliative chemotherapy. However, there are some limitations to our study. First, a selective bias might exist as our study is a retrospective analysis. Second, the incidence of patients with aTE is low, which might diminish the statistical power in multivariate analysis. Third, we only included patients with symptomatic ischemic stroke, which were confirmed by clinical presentation and imaging studies. Our study did not include patients who were too sick to undergo imaging, therefore, the true incidence of aTE in our patient cohort may have been underestimated. Fourth, After reviewing the medical records of cases included in this study, it would be difficult to come up with a fair and accurate measurement to determine if the HTN, CAD, or AF was under control, and as such it was not possible to evaluate the relationship between the severities of these illnesses and aTE in our study. Fifth, we were not able to extrapolate the etiology of these aTE events, i.e., these events were the result of long standing plaques or acute thrombotic events by reviewing the medical records. Finally, our study did not include other parameters, such as D-dimer, fibrinogen, or C-reactive protein, that was found to be associated aTE in cancer patients [18,34], for analysis as it was an inherited limitation of our retrospective study. Further prospective research is necessary to overcome these limitations.

## 4. Patients and Methods

### 4.1. Patient Selection

Medical records of aTE events in patients after initiation of palliative chemotherapy for newly diagnosed unresectable or metastatic pancreatic cancer from 2010 to 2016 at the four affiliated hospitals of Chang Gung Memorial Hospital in Taiwan were retrospectively reviewed. All patients with either pathologically or images diagnosed primary pancreatic cancer receiving palliative chemotherapy were included in this study. Exclusion criteria were as follows: patients with recurring tumors following radical surgery, histological type with neuroendocrine cancer, or concurrent other active malignancy. Finally, a total of 838 consecutive patients were enrolled for analysis. Because mesentery ischemia and peripheral arterial occlusion disease are rare and there was none of these events in our study. We only included ischemic stroke and acute myocardial infarction as an aTE event [10]. Myocardial infarction was confirmed using the third universal definition, including increased levels of cardiac enzymes combined with either clinical symptoms of angina, abnormal electrocardiogram, or identification of thrombus by coronary angiography [35]. Ischemic stroke was defined as a sudden or rapid onset of neurological deficit that was confirmed by a neurologist and neuroimaging with either computed tomography scan or magnetic resonance imaging. Six patients had a diagnosis of vTE and all received low-molecular-weight heparin before the occurrence of aTE events. vTE events were not included in the current analysis. The clinical characteristics of all 838 patients were analyzed to identify independent predictors of aTE and survival outcome. This study was approved by the institutional review boards of all the CGMH branches at 22 November 2017 (ethic code: 201701796B0) and has been conducted in compliance with the Helsinki Declaration (1996).

### 4.2. Data Collection

The patient’s demographic and clinical data including age, sex, body mass index (BMI), Eastern Cooperative Oncology Group performance status (ECOG PS), pre-existing comorbidities by modified Charlson comorbidity index (CCI) [36], anatomic location of the primary cancer, tumor stage, presence of drainage for obstructive jaundice, serum CEA and CA19-9 levels, pre-treatment parameters of complete blood count and biochemistry from peripheral blood, organ of metastatic site, and regimens of chemotherapy were recorded by their primary care physician using a prospectively formulated electronic data form from our previous study. All included patients were followed until death or 31 December 2017. Overall survival (OS) and cumulative incidence of aTE were calculated from the initiation of chemotherapy until the date of death from any cause and occurrence of aTE, respectively. All dates of death were obtained from either the Institutional Cancer Registry or the National Registry of Death database in Taiwan.

### 4.3. Statistical Analysis

Basic demographic data were summarized as n (%) for categorical variables, and median with range or 95% confidence interval (CI) for continuous variables. The parameters of blood tests were analyzed using cutoff values of the median in our study. All the variables in the univariate analysis with *p*-values <0.10 were further analyzed using multivariate analysis. Risk factors for occurrence of aTE were examined by univariate and multivariate logistic regression analysis. All patients were further stratified by the numbers of independent risk factors for aTE they presented with for comparison to the cumulative incidence curve. Survival outcome and cumulative incidence of aTE were calculated according to the Kaplan–Meier method. Log-rank tests were used to determine significant differences between the survival curves. SPSS 17.0 software (SPSS Inc., Chicago, IL, USA) was used for statistical analysis. All statistical assessments were two sided, and a *p*-value < 0.05 was considered statistically significant.

## 5. Conclusions

Patients with pancreatic cancer undergoing palliative chemotherapy have a substantially increased risk of aTE. aTE was a poor prognostic factor and the occurrence of aTE accelerated the ultimate death of patients. Our study identified four variables, including stage IV disease, hypertension, AF, and high AST level (>34 μ/L), as the independent predictors for aTE in pancreatic cancer. A predictive model based on the number of risk factors presented by patients showed those presenting with 3 or 4 variables had the highest risk for the development of aTE. Further study should aim to externally validate our findings to evaluate the performance of this predictive model.

## Figures and Tables

**Table 1:** Basic patient characteristics (*n* = 838).

Variable	Category	Value
Age, years (range)	median	62 (23–89)
Gender, *n* (%)	male	474 (59.3)
	female	364 (40.7)
BMI, kg/m^2^ (range)	median	23 (13.0–36.2)
ECOG PS	0–1	597 (71.2)
	2	206 (24.6)
	3	35 (4.2)
Smoking history, *n* (%)	yes	306 (36.5)
Charlson comorbidity index, *n* (%)	0	227 (27.1)
	1	292 (34.8)
	2	193 (23.0)
	3	102 (12.2)
	4	19 (2.3)
	5	5 (0.6)
Comorbidity, *n* (%)	diabetic mellitus	313 (37.4)
	hypertension	332 (39.6)
	cerebrovascular disease	30 (3.6)
	coronary artery disease	52 (6.2)
	arrhythmia	13 (1.6)
Tumor site of pancreas, *n* (%)	head	343 (40.9)
	body	148 (17.7)
	tail	171 (20.4)
	overlapping	176 (21.0)
Tumor stage, *n* (%)	III	183 (21.8)
	IV	655 (78.2)
Tumor grade, *n* (%)	well to moderate	93 (11.1)
	poorly	92 (11.0)
	unclassified or unknown	653 (77.9)
Presence with obstructive jaundice under drainage, *n* (%)	yes	272 (32.5)
	no	566 (67.5)
Metastatic organ, *n* (%)	liver	438 (52.3)
	peritoneum	239 (28.5)
	lymph nodes	150 (17.9)
	lung	98 (11.7)
Laboratory data, median (range)	Hemoglobin, g/dL	12.3 (3.6–17.4)
	Leukocyte count, 10^9^/L	7600 (1400–77,000)
	Platelet count, 10^9^/L	221 (23–500)
	Albumin, g/dL	3.8 (1.9–4.5)
	AST, μ/L	34 (10–954)
	Alkaline phosphatase, IU/L	110 (10–2688)
	CEA, ng/mL	5.3 (0.3–50,000)
	CA19-9, μ/mL	780 (0.5–50,000)
Use of chemotherapy agent, *n* (%)	Gemcitabine	792 (94.5)
	Platins	433 (51.7)
	S-1	335 (40.0)
	5-fluorouracil	257 (30.7)
	Irinotecan	17 (2.0)
	Erlotinib	14 (1.8)
	Nab-paclitaxel	12 (1.4)
Patients with aTE		42 (5.0)
Type of aTE	ischemic stroke	36 (4.3)
	myocardial infarction	6 (0.7)

**Table 2:** Univariate and multivariate analysis for prediction of aTE.

Variable	Category	ATE No/Total No (%)	Univariate Analysis			Multivariate Analysis		
			HR	95% CI	*p*	HR	95% CI	*p*
BMI, kg/m^2^	≤23	17/474 (3.6)	1			1		
	>23	25/364 (6.9)	1.98	1.05–3.73	0.037	1.69	0.87–3.23	0.12
ECOG PS	0–2	38/803 (4.7)	1			1		
	3	4/35 (11.4)	2.6	0.87–7.73	0.086	1.76	0.52–5.97	0.37
Hypertension	no	14/506 (2.8)	1			1		
	yes	28/332 (8.4)	3.24	1.68–6.25	<0.001	2.74	1.37–5.47	0.004
Cerebrovascular disease	no	37/808 (4.6)	1			1		
	yes	5/30 (16.7)	4.17	1.51–11.5	0.006	1.98	0.62–6.34	0.25
Coronary artery disease	no	38/786 (4.8)	1					
	yes	4/52 (7.7)	1.64	0.56–4.79	0.37			
Arrhythmia	no	39/825 (4.7)	1			1		
	yes	3/13 (23.1)	6.05	1.60–22.9	0.008	4.77	1.10–20.7	0.037
Tumor stage, 7th AJCC	III	3/183 (1.6)	1			1		
	IV	39/655 (6.0)	3.80	1.16–12.4	0.027	4.12	1.24–13.6	0.021
Aspartate transaminase, μ/L	≤34	16/450 (3.6)	1			1		
	>34	26/388 (6.7)	1.98	1.05–3.75	0.036	1.97	1.02–3.78	0.043

BMI, body mass index; ECOG PS, Eastern Cooperative Oncology Group performance status; AJCC, American Joint Committee on Cancer; CEA, Carcinoembryonic Antigen; CA19-9, carbohydrate antigen 19-9; AST, aspartate transaminase.
